# Intracellular islatravir-triphosphate half-life supports extended dosing intervals

**DOI:** 10.1128/aac.00458-24

**Published:** 2024-08-06

**Authors:** Xiaowei Zang, Wendy Ankrom, Walter K. Kraft, Ryan Vargo, S. Aubrey Stoch, Marian Iwamoto, Randolph P. Matthews

**Affiliations:** 1 Merck & Co., Inc., Rahway, New Jersey, USA; 2 Department of Pharmacology, Physiology & Cancer Biology, Thomas Jefferson University, Philadelphia, Pennsylvania, USA; Providence Portland Medical Center, Portland, Oregon, USA

**Keywords:** nucleoside reverse transcriptase, islatravir, half-life, pharmacokinetics, human immunodeficiency virus

## Abstract

Antiretroviral therapy has substantially reduced morbidity, mortality, and disease transmission in people living with HIV. Islatravir is a nucleoside reverse transcriptase translocation inhibitor that inhibits HIV-1 replication by multiple mechanisms of action, and it is in development for the treatment of HIV-1 infection. In preclinical and clinical studies, islatravir had a long half-life (t_½_) of 3.0 and 8.7 days (72 and 209 hours, respectively); therefore, islatravir is being investigated as a long-acting oral antiretroviral agent. A study was conducted to definitively elucidate the terminal t_½_ of islatravir and its active form islatravir-triphosphate (islatravir-TP). A single-site, open-label, non-randomized, single-dose phase 1 study was performed to evaluate the pharmacokinetics and safety of islatravir in plasma and the pharmacokinetics of islatravir-TP in peripheral blood mononuclear cells after administration of a single oral dose of islatravir 30 mg. Eligible participants were healthy adult males without HIV infection between the ages of 18 and 65 years. Fourteen participants were enrolled. The median time to maximum plasma islatravir concentration was 1 hour. Plasma islatravir concentrations decreased in a biphasic manner, with a t_½_ of 73 hours. The t_½_ (percentage geometric coefficient of variation) of islatravir-TP in peripheral blood mononuclear cells through 6 weeks (~1008 hours) after dosing was 8.1 days or 195 hours (25.6%). Islatravir was generally well tolerated with no drug-related adverse events observed. Islatravir-TP has a long intracellular t_½_, supporting further clinical investigation of islatravir administered at an extended dosing interval.

## INTRODUCTION

Human immunodeficiency virus (HIV) remains a significant global health concern, with approximately 39 million people living with HIV and 1.3 million new infections occurring in 2022 (1). The use of current antiretroviral therapy (ART) has achieved substantial reduction in the transmission of HIV and its related morbidity and mortality, such that the life expectancy of people living with HIV is approaching that of the general population (2). However, novel antiretroviral agents with the potential for use at extended dosing intervals that are safe and well-tolerated may improve patient adherence to a drug regimen and provide additional dosing options for patients, further improving the lifelong care required for people living with HIV (3).

**Fig 1:**
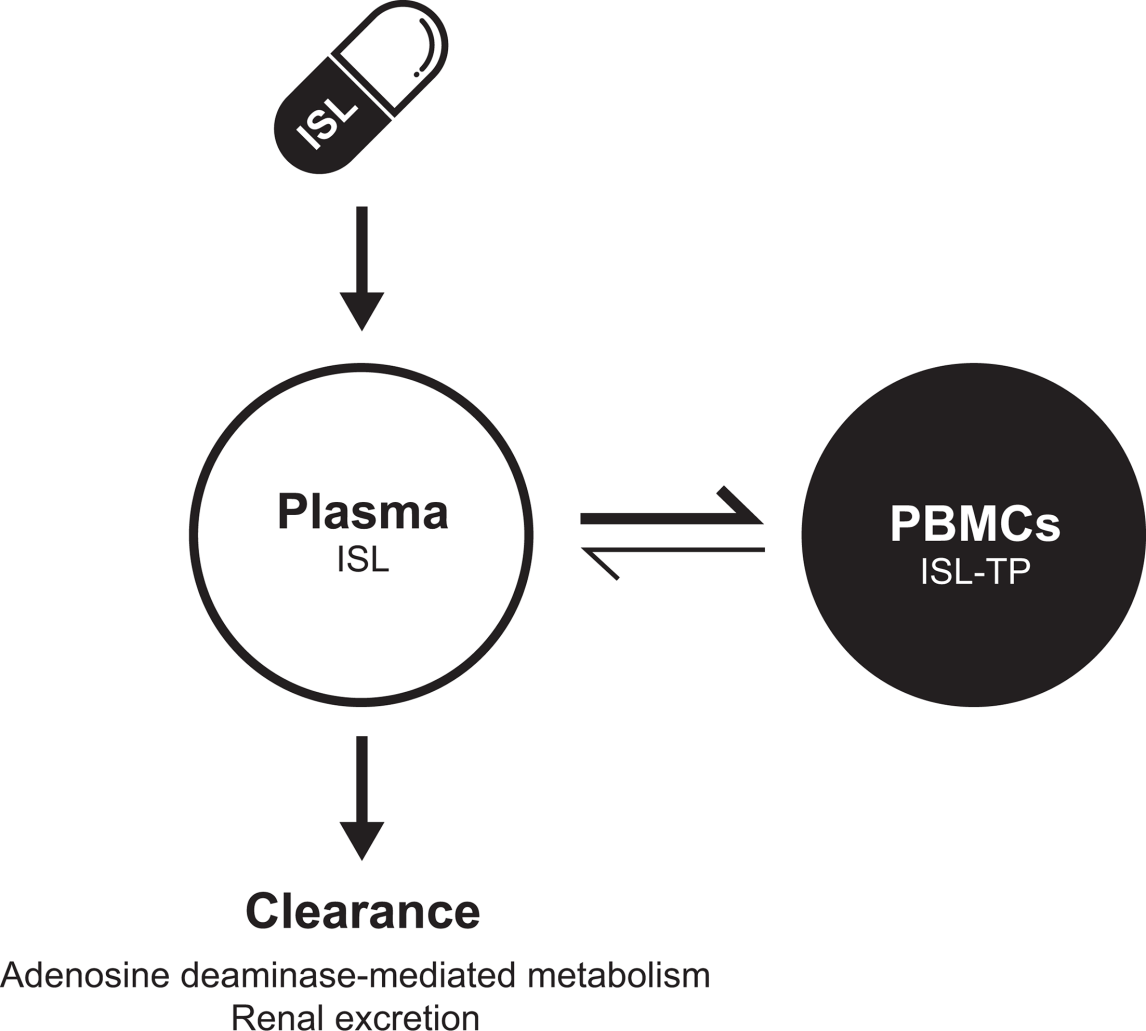
Schematic of islatravir PK pathways. Islatravir, like other deoxyribonucleosides, initially undergoes phosphorylation by deoxycytidine kinase (8). Subsequent phosphorylation steps are likely catalyzed by adenylate kinase and nucleoside diphosphate kinase—pathways shared by other deoxyribonucleosides and related analogs. Islatravir-TP, islatravir triphosphate; PBMC, peripheral blood mononuclear cell; PK, pharmacokinetics.

Islatravir is a nucleoside reverse transcriptase translocation inhibitor (NRTTI) in clinical development for the treatment of HIV-1 infection (4–6). Islatravir inhibits HIV-1 reverse transcriptase by multiple mechanisms of action (4, 5, 7–9), inducing immediate and delayed chain termination (6, 10). When taken up into target cells (CD4+ T cells), islatravir is converted to its pharmacologically active triphosphate form (islatravir-TP) by endogenous intracellular kinases (Fig. 1) (7). In preclinical animal studies (11–13) and several clinical trials (6, 14, 15), islatravir showed antiviral activity against HIV-1 *in vitro* (including resistant variants). The current clinical development program for islatravir is focused on the treatment of HIV-1 infection, including phase 3 studies evaluating a once-daily oral dose in combination with doravirine and a phase 2 study evaluating a once-weekly oral dose in combination with lenacapavir (16).

The pharmacokinetics (PK) profile of plasma islatravir is well characterized and exhibits dose-proportional PK for a wide range of doses (0.25–400 mg) (3, 6, 7, 17). After single-dose oral administration, islatravir circulates in plasma where it is absorbed intracellularly (Fig. 1). Peak islatravir plasma concentrations are achieved 30–60 minutes after dosing (3, 7), and peak islatravir-TP concentrations are achieved 6–24 hours after dosing (3, 6, 7), as measured in peripheral blood mononuclear cells (PBMCs). Islatravir is eliminated by renal excretion of the parent drug and by adenosine deaminase-mediated metabolism (Fig. 1) to the major circulating islatravir metabolite, 4′-ethynyl-2-fluoro-2′deoxyinosine (M4). M4 cannot be phosphorylated and is inactive; it is also renally excreted (17, 18). Plasma concentrations of islatravir decrease in a biphasic manner, with an initial rapid phase and a slow terminal phase (3, 6, 7). This slow terminal phase may be due to redistribution of the parent drug from cells back into the plasma after initial clearance, as would be expected if islatravir-TP is dephosphorylated back into islatravir in cells and exits, returning to the plasma (Fig. 1) (3). Initial estimates of the intracellular half-life (t_½_) of islatravir-TP range from 79 to 209 hours (3, 6); this wide range is likely due to inconsistencies in handling of PBMCs (7, 19) and to short duration of PK sampling in previous studies (6, 17). Although the long t_½_ of islatravir-TP indicates that islatravir may be suitable for extended-duration dosing regimens (3, 7, 17), dose selection for extended intervals is dependent on a reliable estimate of the t_½_ of islatravir and islatravir-TP. We conducted a study to evaluate the apparent t_½_ of plasma islatravir and intracellular islatravir-TP after administration of a single oral dose of islatravir 30 mg.

## RESULTS

### Study population

Fourteen participants were randomized and administered a single oral dose of islatravir 30 mg. Participant demographics and baseline characteristics are described in Table 1.

**TABLE 1:** Baseline characteristics of the study population^*a*^

Characteristic	All participants (*N* = 14)
Sex, male, *n* (%)	14 (100)
Age, median (range), years	45.5 (19–56)
Race, *n* (%)	
Black or African American	12 (85.7)
White	2 (14.3)
Ethnicity, *n* (%)	
Hispanic or Latino	1 (7.1)
Not Hispanic or Latino	12 (85.7)
Unknown	1 (7.1)
BMI, mean (SD), kg/m^2^	29.1 (2.9)

^*a*^ BMI, body mass index.

### Pharmacokinetics

**Fig 2:**
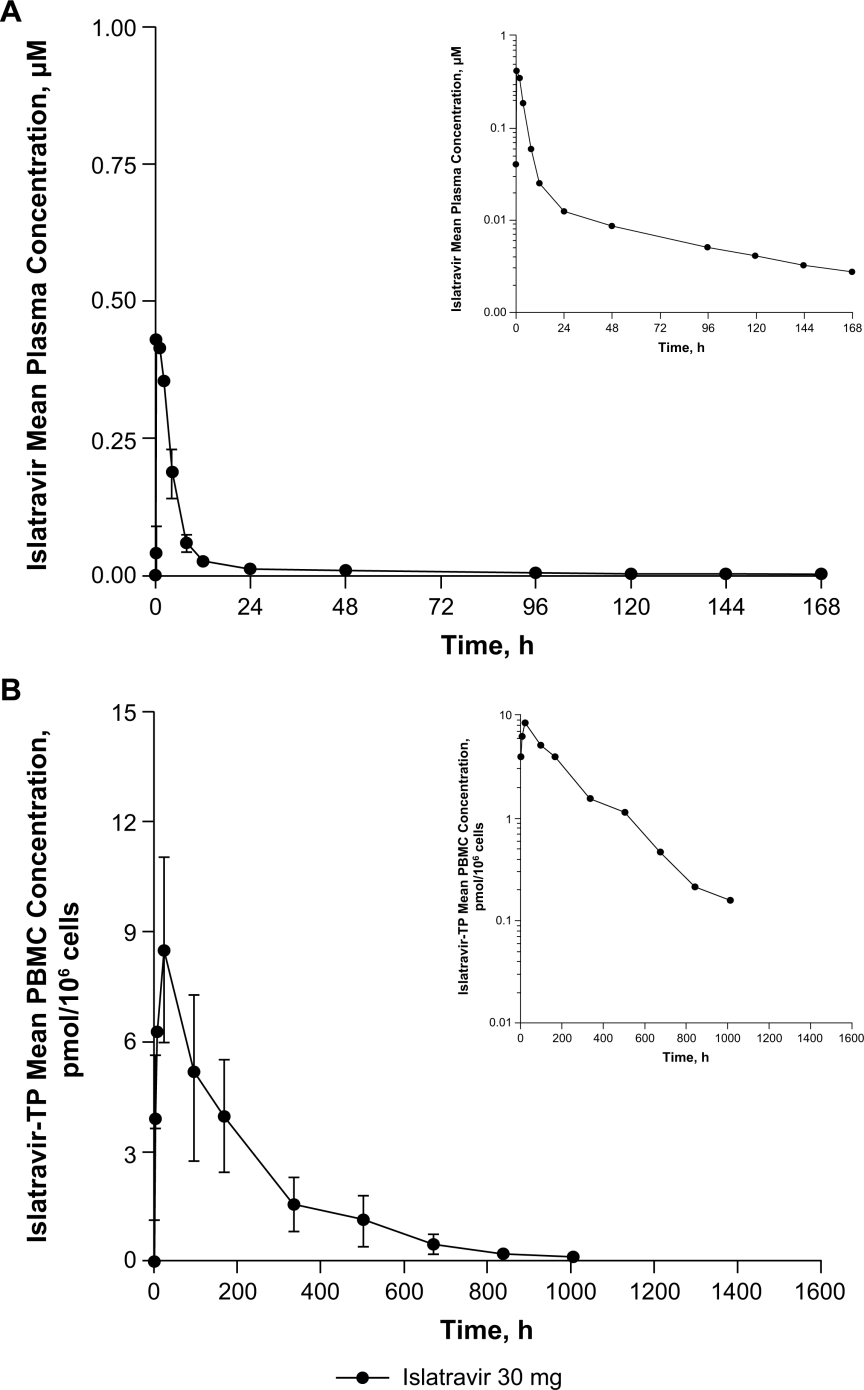
Arithmetic mean (± SD) plasma and PBMC concentration versus time profiles of (**A**) islatravir plasma and (**B**) islatravir-TP PBMC after administration of a single oral dose of islatravir 30 mg to healthy participants (*N* = 14); inset: semi-log scale. Islatravir-TP, islatravir-triphosphate; PBMC, peripheral blood mononuclear cell.

The mean islatravir plasma concentration–time profile is shown in Fig. 2A, and PK parameter values are shown in Table 2. The median time to maximum concentration (T_max_) was 1 hour after treatment with islatravir 30 mg (Table 2). Plasma islatravir concentrations decreased in a biphasic manner, with a t_½_ of 73 hours (Fig. 2A; Table 2).

**TABLE 2:** Summary geometric mean of plasma islatravir and PBMC islatravir-TP PK in participants after administration of a single dose of islatravir 30 mg^*a*^

	PK parameter	GM (%GCV) (*N* = 14)
Islatravir (plasma)	C_max_, µM	0.532 (45.2)
	T_max_, h^*b*^	1.00 (0.48–2.03)
	AUC_0–inf_, h·µM	3.08 (20.2)
	AUC_0–168_, h·µM	2.80 (20.4)
	t_½_, h	73.0 (17.3)
Islatravir-TP (PBMCs)	AUC_0–inf_, h·pmol/10^6^ cells	1700 (38.6)
	AUC_0–168_, h·pmol/10^6^ cells	882 (36.8)
	AUC_0–1008_, h·pmol/10^6^ cells	1660 (38.7)
	C_168_, pmol/10^6^ cells	3.68 (44.2)
	t_½_, h	195 (25.6)

^*a*^ AUC_0–168_, area under the concentration–time curve from before dose to 168 hours after dosing; AUC_0–1008_, area under the concentration–time curve from before dosing to 1008 hours (6 weeks) after dosing; AUC_0–inf_, area under the concentration–time curve from before dosing to infinity; C_168_, concentration at 168 hours after dosing; C_max_, maximum plasma concentration; GCV, geometric coefficient of variation; GM, geometric mean; PBMC, peripheral blood mononuclear cell; PK, pharmacokinetics; t_½_, apparent terminal half-life; T_max_, time to maximum concentration.

^*b*^ Median (range).

The mean islatravir-TP PBMC concentration–time profile is shown in Fig. 2B, and PK parameter values are shown in Table 2. The terminal t_½_ geometric coefficient of variation (%GCV) of islatravir-TP in PBMCs through 6 weeks (~1008 hours) after dosing was 8.1 days or 195 hours (25.6%) (Table 2). There was no evidence of a secondary terminal phase for islatravir-TP in PBMCs (Fig. 2B).

### Safety

A single oral dose of islatravir 30 mg was well tolerated. Eight participants (57.1%) reported adverse events (AEs), but none was determined to be drug-related. No AE was reported by >1 participant. All AEs were grade 1 or 2, and most AEs resolved by the end of the study.

No clinically meaningful changes in electrocardiograms, vital signs, or laboratory values were observed after islatravir dosing. No serious AEs were reported in the current study.

## DISCUSSION

In this open-label, non-randomized, single-dose, phase 1 study, the safety and PK profiles of islatravir in plasma and islatravir-TP in PBMCs were assessed in healthy adult male participants without HIV infection to definitively determine the intracellular t_½_ of the long-acting active metabolite islatravir-TP. The t_½_ of islatravir in plasma was 3.0 days (73 hours), and the t_½_ of islatravir-TP in PBMCs was 8.1 days (195 hours), noted in previous studies (3, 6). The observed t_½_ of islatravir-TP is also consistent with the degree of accumulation noted in previous studies (3, 7). The longer sampling period (~8 half-lives) and optimized process of handling samples in the current study resulted in a more accurate assessment of islatravir-TP t_½_ than in previous studies (3, 6, 7, 17). In previous studies, PBMC sample collection was conducted for ≤2 months. Due to islatravir-TP concentrations being variable and highly sensitive to sample handling methods, there was uncertainty as to whether there may be a longer terminal phase in the islatravir-TP concentration–time curve. In the current study, the intent was to collect PBMC samples up to 20 weeks after dosing to definitively determine the terminal t_½_ of islatravir-TP.

The plasma PK profile of islatravir in the current study was consistent with what was seen previously (3, 6, 7, 17). Islatravir is rapidly absorbed and cleared from plasma in a biphasic manner. Islatravir is rapidly cleared from plasma and redistributed as intracellular islatravir-TP, which is gradually dephosphorylated and returns as islatravir to the plasma. Islatravir-TP was cleared from PBMCs in an approximately linear manner over the 6 weeks of the PK analysis.

Because CD4^+^ T cells are the primary site of HIV-1 infection, replication, and latency (20, 21), the use of ARTs (22) that persist at therapeutic concentrations in lymphoid cells could potentially have a positive impact on long-term treatment outcomes (21). Measurement of ARV levels in PBMCs is a suitable proxy for understanding ARV PK in CD4+ T cells. The long intracellular t_½_ of islatravir-TP in PBMCs makes extended dosing intervals possible, potentially helping to overcome barriers to adherence to ART that are related to dosing frequency. Decreasing total lymphocyte and CD4+ T cell counts were observed across several phase 2 and 3 clinical studies with islatravir (16). Due to these findings, new clinical programs with islatravir 0.25 mg once daily and 2 mg once weekly in combination with other ARVs were initiated (23). In the current study, no meaningful changes were seen in lymphocyte counts; however, the present study had a very limited sample size with only a single dose of islatravir 30 mg administered.

Adherence to ART is a challenge for people living with HIV, and it is hampered by many factors, such as long-term tolerability, treatment fatigue, and the potential for drug–drug interactions (24, 25). Long-acting therapies for the treatment and prevention of chronic disorders, such as cardiovascular disease, cancer, kidney disease, and diabetes have helped to improve adherence, compared with treatments that require more frequent administration, leading to better clinical outcomes and retention in care (26). This is especially important for people living with HIV; eligible patients willing to switch to a long-acting ARV drug are particularly interested in the improved convenience, freedom, confidentiality, and emotional benefits of not being reminded each day of their HIV status through daily pill use (24).

The key limitations of this study are a small sample size and short time of exposure to a single dose of islatravir. Furthermore, only male participants were enrolled to decrease demographic variables. This study was part of a larger investigation with another compound, which did not have reproductive and toxicity data, and, consequently, inclusion of female participants was limited to those of non-childbearing potential. Therefore, although women were not strictly excluded, only male participants were enrolled due to the small pool of potential female participants of non-childbearing potential. Additional studies will be necessary to confirm the duration of t_½_ in a larger population of people living with HIV. Previously, it was found that the PK profile of islatravir in people living with HIV (6) was similar to the PK profile observed in participants without HIV (7), suggesting that the t_½_ observed in the current study is generalizable to people living with HIV. The t_½_ observed in the current study is consistent with observations in multiple previous studies; the greater length of time for data collection in the current study allows for a more accurate estimation of the t_½_ moving forward in the clinical development of islatravir for extended-dosing regimens.

### Conclusion

The results of the current study showed that, after a single oral dose of islatravir 30 mg, the intracellular t_½_ of islatravir-TP, the active metabolite of islatravir, was 8.1 days (195 hours) over a PK sampling period of 6 weeks. No new safety signals were identified. Further development of islatravir with extended dosing intervals for the treatment of HIV-1 is warranted and is substantiated by the results of the current study.

## MATERIALS AND METHODS

### Study design

A single-site, open-label, non-randomized, single-dose, phase 1 study was conducted to evaluate the PK of islatravir in the plasma and of islatravir-TP in PBMCs after administration of a single oral dose of islatravir 30 mg (protocol MK-8558–004, period 3).

### Participants

The participants were healthy adult males without HIV infection between the ages of 18 and 65 years without HIV infection and with a body mass index (BMI) between 18 and 33 kg/m^2^. Key inclusion and exclusion criteria are described in the supplemental material.

### Study procedures

All participants were housed overnight at the clinical research center before study drug dosing and remained there until 24 hours after dosing. After an overnight fast of ≥10 hours, the participants were administered a single oral dose of islatravir 30 mg (3 × 10-mg capsules) with 240 mL of water.

### Study assessments

Blood was collected for plasma islatravir PK analysis on Day 1 and at 0.25, 0.5, 1, 2, 4, 8, 12, 24, 48, 96, 120, 144, and 168 hours (1 week) after dosing. Blood was collected for PBMC islatravir-TP PK analysis on Day 1 and at 4, 12, 24, 96, 168, 336, 504, 672, 840, and 1008 hours (6 weeks) after dosing.

Plasma and PBMC samples were assayed for islatravir or islatravir-TP, respectively, using a validated high-performance liquid chromatography–tandem mass spectrometry method. The analytical range of the plasma islatravir assay was 100–100,000 pg/mL, and the analytical range of the PBMC islatravir-TP assay was 0.1–40.0 ng/mL. At 15 minutes after dosing, islatravir concentrations were below the limit of quantification in two participants; at 840 hours after dosing, islatravir-TP concentrations were below the limit of quantification in one participant. Islatravir in plasma and islatravir-TP concentrations below the limit of quantification were treated as zero. PBMC cell counts (per 10^6^ cells) were estimated using a hemocytometer, and the conversion from islatravir-TP ng/mL to micromolar was determined using the molecular weight of islatravir-TP at 533.19 g/mol. The conversion from micromolar to picomole per 10^6^ cells was made using the standard assumption that 1 PBMC has an approximate volume of 0.2 pL (27, 28).

Safety assessments—which included vital signs (temperature, heart rate, respiratory rate, and blood pressure), electrocardiograms, and clinical laboratory assessments—were carried out before dosing on Days 0, 2, and 8. AEs were monitored throughout the study period.

### Statistics

All PK parameters were calculated by non-compartmental analyses using Phoenix WinNonlin software (version 8.1; Certara, Princeton, NJ, USA). For each PK parameter, individual values were natural log-transformed and evaluated using a linear mixed-effects model. A 90% CI was constructed for the difference in least squares means on the log scale; exponentiating the log-scale 90% CI provided a 90% CI for the true geometric mean ratio (GMR) for the PK parameters. GMRs and their corresponding 90% CIs for all area under the concentration–time curve (AUC) parameters and maximum concentrations (C_max_)/concentration at 168 hours (C_168_) were estimated. Descriptive statistics (non-model-based) were calculated using SAS (version 9.4; SAS Institute, Cary, NC, USA) provided for each PK parameter, including arithmetic mean and standard deviation, median and range, and geometric mean (GM) and %GCV.

## Data Availability

The data sharing policy, including restrictions, of Merck Sharp & Dohme LLC, a subsidiary of Merck & Co., Inc., Rahway, NJ, USA, is available at http://engagezone.msd.com/ds_documentation.php. Requests for access to the clinical study data can be submitted through the Engage Zone site or via email to dataaccess@merck.com

## SUPPLEMENTAL MATERIAL

Supplemental material aac.00458-24-s0001.pdf Inclusion and exclusion criteria.

ASM does not own the copyrights to Supplemental Material that may be linked to, or accessed through, an article. The authors have granted ASM a non-exclusive, world-wide license to publish the Supplemental Material files. Please contact the corresponding author directly for reuse.
