# Supplementary material for: Intracellular islatravir-triphosphate half-life supports extended dosing intervals
Source: Antimicrob Agents Chemother. 2024 Aug 6;68(9):e00458-24. doi: 10.1128/aac.00458-24 (PMC11382622; doi:10.1128/aac.00458-24)
Supplement: Supplemental material — Inclusion and exclusion criteria. [file aac.00458-24-s0001.pdf]

Supplemental Materials

Inclusion criteria

As stated in the protocol, participants were eligible to be included in the study if the participant:

1. Was in good health based on medical history, physical examination, vital sign measurements, and ECG performed before to randomization
2. Was in good health based on laboratory safety tests obtained at the screening visit and before administration of the initial dose of study drug
3. Had a BMI ≥18.5and ≤33 kg/m^2
4. Was male or female, from 18 years to 65 years of age inclusive, at the time of signing the informed consent
    - For male participants, no measures were needed, but contraceptive use by men was consistent with local regulations regarding the methods of contraception for those participating in clinical studies and ≥1 of the following conditions applied:
        - Was abstinent from heterosexual intercourse as their preferred and usual lifestyle (abstinent on a long term and persistent basis) and agree to remain abstinent or was using an acceptable contraceptive method unless confirmed to be azoospermic (vasectomized or secondary to medical cause) as detailed below:
            - Agree to use a male condom plus partner use of an additional contraceptive method when having penile-vaginal intercourse

with a women of non-child bearing potential who was not pregnant at the time of study

- A female participant was eligible to participate if she was of non-child bearing potential

5. The participant (or legally acceptable representative) was required to provide documented informed consent/assent for the study, including for future biomedical research

Exclusion Criteria

As stated in the protocol, participants were excluded from the study if the participant:

1. Had a history of clinically significant endocrine, gastrointestinal, cardiovascular, hematological, hepatic, immunological, renal, respiratory, genitourinary, or major neurological (including stroke and chronic seizures) abnormalities or diseases. Participants with a remote history of uncomplicated medical events (eg, uncomplicated kidney stones, defined as spontaneous passage and no recurrence in the past 5 years, or childhood asthma) were enrolled in the study at the discretion of the investigator

2. Was mentally or legally incapacitated; had significant emotional problems at the time of the screening visit or that were expected during the conduct of the study; or had a history of a clinically significant psychiatric disorder of the past 5 years. Participants who had situational depression were enrolled in the study at the discretion of the investigator

3. Had a history of cancer (malignancy) with the following exceptions:

- Adequately treated nonmelanomatous skin carcinoma or carcinoma in situ of the cervix
- Other malignancies which were successfully treated with appropriate follow-up and, therefore, in the opinion of the investigator and with agreement of the sponsor, was unlikely to recur for the duration of the study, (eg, malignancies which were successfully treated 10 years before the screening visit)

4. Had an estimated CrCl ≤ 70 mL/min based on the C-G Equation
    - At the discretion of the investigator, a measured creatinine clearance, as determined by a 24-hour urine collection, was used in place of, or in conjunction with, the estimate of the creatinine clearance
5. Had a history of significant multiple and/or severe allergies (eg, food, drug, latex allergy) or had had an anaphylactic reaction or significant intolerability (ie, systemic allergic reaction) to prescription or nonprescription drugs or food
6. Was positive for hepatitis B surface antigen, hepatitis C antibodies, or HIV
7. Had major surgery or donated or lost 1 unit of blood (approximately 500 mL) within 4 weeks before the screening visit
8. Was unable to refrain from or anticipated the use of any medication, including prescription and nonprescription drugs or herbal remedies beginning approximately 2 weeks (or five half-lives) before administration of the initial dose of study drug, throughout the study (including washout intervals between treatment periods) until the poststudy visit

9. If female, was taking hormone replacement therapy within the 60 days preceding screening
10. Had participated in another investigational study within 4 weeks (or five half-lives, whichever was greater) before the screening visit. The window was derived from the date of the last visit in the previous study
11. Had a clinically significant abnormality on the ECG performed at the pre-study (screening) visit and/or prior to administration of the initial dose of study drug
12. Was unwilling to comply with the study restrictions
13. Was a smoker and/or had used nicotine or nicotine-containing products (eg, nicotine patch and electronic cigarette) within 3 months of screening
14. Consumed more than three glasses of alcoholic beverages (one glass was approximately equivalent to beer [354 mL/12 oz], wine [118 mL/4 oz], or distilled spirits [29.5 mL/1 oz]) per day. Participants who consumed 4 glasses of alcoholic beverages per day were enrolled at the discretion of the investigator
15. Consumed excessive amounts, defined as more than six servings (one serving was approximately equivalent to 120 mg of caffeine) of coffee, tea, cola, energy drink, or other caffeinated beverage per day
16. Was a regular user of cannabis or any illicit drug or had a history of drug (including alcohol) abuse within approximately 2 years before enrollment. Participants were required to have a negative urine drug screening result before randomization

17. Presented any concern by the investigator regarding safe participation in the study or for any other reason the investigator considered the participant inappropriate for participation in the study
18. Was or had an immediate family member (eg, spouse, parent/legal guardian, sibling, or child) who was investigational site or sponsor staff directly involved with the current study
